# pTrimmer: An efficient tool to trim primers of multiplex deep sequencing data

**DOI:** 10.1186/s12859-019-2854-x

**Published:** 2019-05-10

**Authors:** Xiaolong Zhang, Yanyan Shao, Jichao Tian, Yuwei Liao, Peiying Li, Yu Zhang, Jun Chen, Zhiguang Li

**Affiliations:** 1 Center of Genome and Personalized Medicine, Institute of Cancer Stem Cell, Dalian Medical University, Dalian, 116044 Liaoning China; 2 The Second Hospital of Dalian Medical University, 467th Zhongshan Road, Shahekou District, Dalian, 116023 Liaoning China; 3 The Second Affiliated Hospital, School of Medicine, Zhengzhou University, Zhengzhou, 450052 China

**Keywords:** Primer trimming, Target sequencing, Multiplex amplicon sequencing

## Abstract

**Background:**

With the widespread use of multiple amplicon-sequencing (MAS) in genetic variation detection, an efficient tool is required to remove primer sequences from short reads to ensure the reliability of downstream analysis. Although some tools are currently available, their efficiency and accuracy require improvement in trimming large scale of primers in high throughput target genome sequencing. This issue is becoming more urgent considering the potential clinical implementation of MAS for processing patient samples. We here developed pTrimmer that could handle thousands of primers simultaneously with greatly improved accuracy and performance.

**Result:**

pTrimmer combines the two algorithms of k-mers and Needleman-Wunsch algorithm, which ensures its accuracy even with the presence of sequencing errors. pTrimmer has an improvement of 28.59% sensitivity and 11.87% accuracy compared to the similar tools. The simulation showed pTrimmer has an ultra-high sensitivity rate of 99.96% and accuracy of 97.38% compared to cutPrimers (70.85% sensitivity rate and 58.73% accuracy). And the performance of pTrimmer is notably higher. It is about 370 times faster than cutPrimers and even 17,000 times faster than cutadapt per threads. Trimming 2158 pairs of primers from 11 million reads (Illumina PE 150 bp) takes only 37 s and no more than 100 MB of memory consumption.

**Conclusions:**

pTrimmer is designed to trim primer sequence from multiplex amplicon sequencing and target sequencing. It is highly sensitive and specific compared to other three similar tools, which could help users to get more reliable mutational information for downstream analysis.

**Electronic supplementary material:**

The online version of this article (10.1186/s12859-019-2854-x) contains supplementary material, which is available to authorized users.

## Background

Genomic mutation detection has become more and more popular in clinical cancer research [1]. Multiplex amplicon-based deep sequencing is one of the major approaches for mutation detection of specific diseases-associated genes [1–3]. Many algorithms have been developed to identify mutations in cancer-associated genes [2, 4]. The reads from multiplex amplicon-based next-generation sequencing (NGS) usually contain two parts, namely gene-specific primers (i.e., sequence used to amplify the target regions) and region of interest [5]. The contiguous amplicon sequences are usually designed to overlap with each other to cover the whole genes of interest [6]. However, the ineffective oligonucleotide synthesis will introduce errors to primers, which means mapping the untrimmed NGS reads directly to reference genome would lead to unreliable mutation information. Specifically, the synthesis errors of primers would be mistakenly regarded as containing the mutational information, which would increase the value of variant allele frequency (VAF) at the corresponding genomic site, and therefore increasing its probability to be called as a ‘true’ nucleotide variant by the mutation caller software.

Some tools are currently available or compatible with primer trimming, such as cutadapt [7] and AlienTrimmer [8]. These tools work efficiently in removing adapter sequences. But multiplex amplicon sequencing usually has hundreds of or thousands of primer sequences [2]. These tools become inefficient in trimming multiplex primer sequences. BAMClipper, a bam-based primer removing tool, focuses on the detection of insertion and deletion near the edge of region-of-interest [5]. However, the Perl implementation and soft-clipping based primer matching algorithm make it rather low in processing large datasets. Some other tools, such as the technical sequences removing program, Trimmomatic [9], can only trim the specific sequence from 3′-end instead of primer sequences from both 5′-end and 3′-end. The tool of cutPrimers [10] is specially developed to remove multiplex primer sequences, however, the algorithm of regular expression matching and Python implementation result in the low sensitivity, specificity and performance.

In this study, we developed a new tool, pTrimmer, to trim primer sequence from multiplex amplicon sequence data. To increase the sensitivity and specificity, we employed both k-mers algorithm [11] and Needleman-Wunsch algorithms [12]. And the C implementation of the tool ensures its high performance. This tool can be deployed on both Windows and Linux systems. Benchmark analysis with three other tools shows that pTrimmer is highly time-efficient and accurate in primer trimming.

### Implementation

**Fig. 1:**
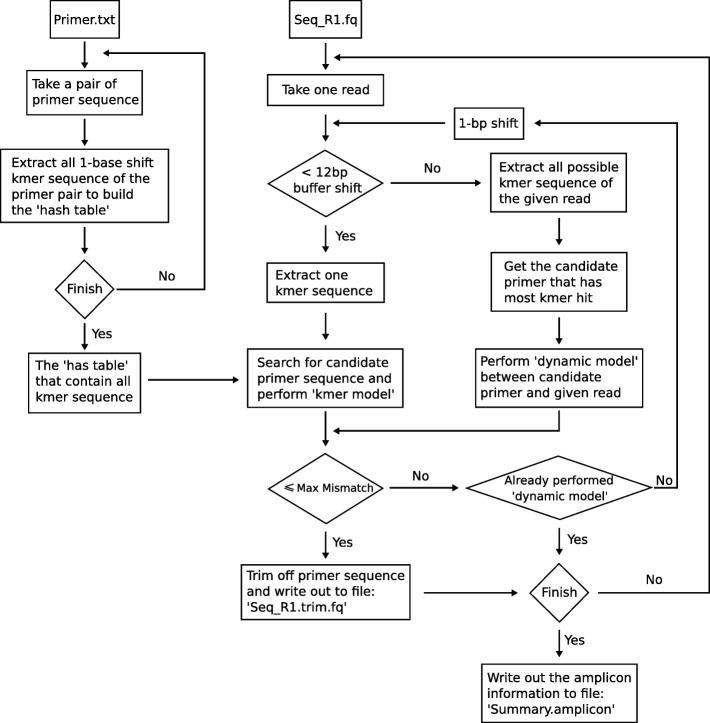
Workflow of pTrimmer. The flowchart shows the details of the program. The program takes a primer sequence file and the two FASTQ files (paired-end sequencing) or one (single-end sequencing) as input. The primer sequence file contains three necessary fields for the forward primer, reverse primer and insert length of amplicon. pTrimmer will first extract all possible 1-base shift kmer sequence of both forward primer and reverse primer to build the ‘hash table’. Kmer sequence will also be extracted from FASTQ read with 1-bp shift each time to search for best candidate primer from ‘hash table’. The 12-bp buffer shift length enable the program has 12 times to find the best primer sequence with ‘k-mers model’. If fails, the program will automatically switch to ‘dynamic model’. The ‘dynamic model’ could extract all possible 1-bp shift kmer sequence and get the best candidate primer that has most kmer hit. Then the program will perform dynamic programing between candidate primer sequence and given read

The pTrimmer workflow (Fig. 1) is implemented with C language and is available to 32-bit/64-bit Windows and Linux platforms. pTrimmer takes fastq (single-end or paired-end) and a set of amplicon primer pairs as input. The method firstly preprocesses the given file to obtain the maximum sequence read length, the maximum primer length and the base quality encoding format (Phred+ 33 or Phred+ 64). Before the processing, pTrimmer loads the amplicon primer sequences into the computer’s RAM and extracts all potential 1-base shift k-mer sequence (default: 8-bp). Then, a ‘hash table’ is constructed to record k-mer sequences and their corresponding indexes, i.e. relative positions, in the primer sequence. To increase the sensitivity and specificity, pTrimmer employs two approaches, named ‘k-mers model’ and ‘dynamic model’, to detect primer sequences within the reads. The ‘k-mers model’ (Additional file 2: Figure S1) takes part of 5′ end of sequence from NGS reads, usually equal to maximum primer length plus 12 bp buffer length, as input and extract all potential 1-base shift k-mer subsequences. Then, all the k-mer subsequences are mapped to the ‘hash table’ (built with amplicon primers) to find all potential candidate primers and the ‘hamming distance’ are calculated between reads and candidate primers to find the best matches. The best match is the primer that has minimum mismatches with the target NGS reads. No scoring scheme was employed, and the search complexity approaches O(1), which leads to extremely fast running time of pTrimmer.

The ‘k-mers model’ isn’t able to properly handle indels in the primer regions of the reads. This shortcoming can be made up by the Needleman-Wunsch algorithm of ‘dynamic model’. pTrimmer prefers to use the ‘k-mers model’ to detect the primer sequence. If failed, it switches to ‘dynamic model’ automatically. The ‘dynamic model’ (Additional file 2: Figure S1) takes all the potential k-mer sequences into consideration and calculates the score of each candidate primer. The candidate primer that has the highest score will perform dynamic programming algorithm with given reads to calculate the ‘edit distance’. The score is the number of k-mer sequence hits per potential primer sequence. And the ‘dynamic programming’ algorithm is performed using the Needleman-Wunsch local algorithm with the parameters of 0 for match, − 1 for mismatch and − 1 for gap extension.

**Fig. 2:**
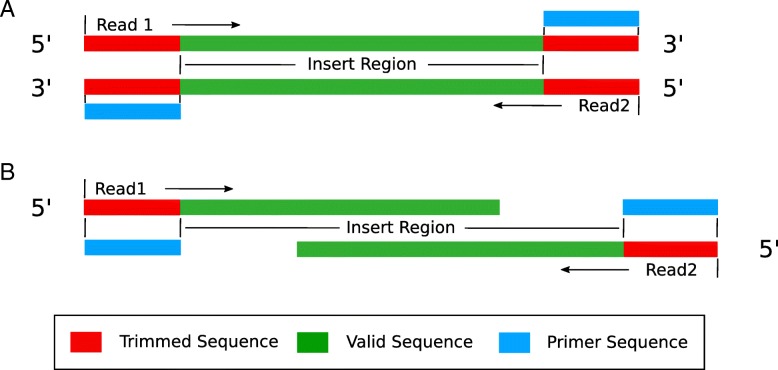
Two conditions of sequencing read. **a** The ‘read-through condition’ has a forward primer (5′ end) and a reverse complementary primer (3′ end) at the right and left ends of both read1 and read2. This situation is pretty popular in liquid biopsy studies, where the amplicon length is around 140 bp due to the confine of cfDNA fragment length while the read length could be 150 bp. **b** The ‘normal condition’ only has forward primer at the 5′ end of reads, both read1 and read2. This occurs when the amplicon length is larger than read length

pTrimmer could handle two conditions of primer trimming, referred to as ‘read-through condition’ (Fig. 2a) and ‘normal condition’ (Fig. 2b). In the ‘read-through condition’, the method firstly detects the 5′ end forward primer sequence and then detects the reverse complementary sequence of reverse primer at 3′ end. Instead, the ‘normal condition’ only detects and removes the 5′ end forward primer. The two conditions are distinguished by the insert length of amplicon. A standard Ubuntu16.04 laptop computer, with 4 cores i7-5500U 2.4GHz and 8 GB RAM, can handle 11 million reads within 37 s, during which the peak memory consumption is no more than 100 MB.

### Results

DNA synthesis could reach the error rate of about 1 in 300–600 bases [13, 14]. To evaluate the effects of primer synthesis error on mutation calling in multiplex amplicon sequencing, we calculated the number of mutations in three sample datasets using the raw reads that include primers or the primer-trimmed counterparts. After primer trimmed, the number of 3892, 9221 and 33,760, potential mutations were found in the sample datasets cfDNA1, cfDNA2 and cfDNA3. With primer region inclusion, the number of potential mutations increased to 8060 (107% increase), 17,378 (88%) and 54,550 (62%), respectively (Additional file 1: Table S1), indicating primer synthesis error could have large impacts on mutation calling. However, clipping primers would shorten the reads, and thus possibly pose difficulties to identify the correct genomic location during alignment. To clear out this concern, we mapped both the raw reads and primer-trimmed reads to reference genome by BWA software. We found averagely 99.7% of primer-trimmed reads were mapped exactly to the same genomic locations as raw reads in the three target sequencing datasets, cfDNA1 (99.8%), cfDNA2 (99.7%) and cfDNA3 (99.6%), indicating the shortened reads contain sufficient information for correct alignment.

As a benchmark, we compared pTrimmer to other three tools (Alientrimmer Cutadapt and cutPrimers) on a Centos7 server, equipped with 32 cores E5–2630 V3 2.4GHz and 128 GB RAM, with multiplex amplicon datasets of cfDNA1–3. All the processing, including the time consumed on primer trimming and the ratio of trimmed reads, are reported in the Table 1. The commands used in the benchmark are listed in the Additional file 1: Command Line. pTrimmer is extremely time-efficient, processing 11 million reads in 35 s, 32 times faster than the second time-efficient program, Alientrimmer (Table 1). This advantage becomes more obvious with the increase of input reads amount. When fed with a fastq of 36 million reads, pTrimmer gets almost 100 times faster than Alientrimmer (Table 1). Moreover, pTrimmer is highly accurate. In most cases, pTrimmer removes and only removes the primer sequences from short reads, leaving the insert sequences for downstream analysis. In the three cfDNA samples, the percentage of ‘precisely trimmed reads’ in total reads averagely reached 92.93% in pTrimmer. This number is 43.83% increased for the secondly ranked program, cutPrimmers.

**Table 1:** Benchmarks of four programs with three multiplex amplicon sequencing datasets. ‘Trimmed Reads’ indicates the reads that had any number of bases removed by the program. ‘Precisely Trimmed Reads’ indicates the reads whose primer sequences were precisely trimmed. K: the number of nucleotides for “k-mer” algorithm. Err: the number or percentage of allowed mismatches for primer searching. See Additional file 1 for the detailed parameters to run each of these programs

Sample (No. of reads)		Alientrimmer	Cutadapt	cutPrimers	
	Parameter	1 thread, K = 9, err = 3	8 threads, err = 10%	8 threads, err = 3	2 threads, k = 9, err = 3
CfDNA1 (11,498,560)	Time of	1137	108,918	2224	35
CfDNA2 (16,694,386)	Running (s)	2511	170,920	3646	47
CfDNA3 (36,119,764)		6099	349,789	7772	62
CfDNA1 (11,498,560)	No. of	2,228,698 (19.28)	11,392,006 (99.07)	8,733,142 (75.95)	10,973,548 (95.43)
CfDNA2 (16,694,386)	Trimmed Reads (%)	3,193,584 (19.13)	16,633,850 (99.64)	12,481,890 (74.77)	16,023,158 (95.98)
CfDNA3 (36,119,764)		5,680,392 (15.73)	35,921,534 (99.45)	24,460,516 (67.72)	32,314,848 (89.47)
CfDNA1 (11,498,560)	No. of	81,143 (0.71)	389,526 (3.39)	7,752,109 (67.42)	10,908,534 (94.87)
CfDNA2 (16,694,386)	Precisely Trimmed Reads (%)	134,647 (0.81)	516,377 (3.09)	11,130,147 (66.67)	5,905,037 (95.27)
CfDNA3 (36,119,764)		236,252 (0.65)	1,003,057 (2.78)	21,581,726 (59.75)	12,024,840 (88.66)

**Fig. 3:**
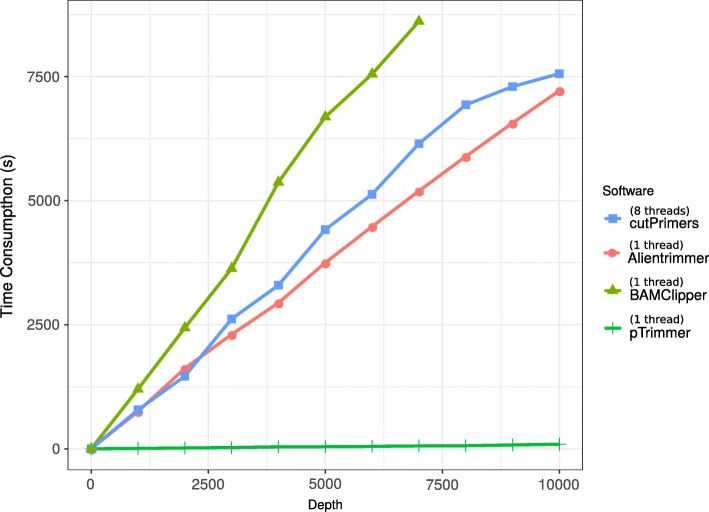
Time consumption of Alientrimmer, cutPrimers pTrimmer and BAMClipper with the increasing depth from 1000× to 10,000×. The results of Cutadapt were excluded due to its huge time consumption. And the results of BAMClipper is the running time of processing the bam files. For better displaying, we run Alientrimmer, BAMClipper and pTrimmer with one thread and run cutPrimers with 8 threads

To have a better view of pTrimmer, we simulate 10 MA datasets with a depth of 1000 × − 10,000× to study the time consumption of these tools. The results show that pTrimmer has a notably higher efficiency (Fig. 3). Varying parameters of tolerant mismatch number are applied to a simulation dataset (100×) to explore the performance of pTrimmer. The Table 2 shows that pTrimmer is capable of achieving ultra-high sensitivity (99.96%) and accuracy (99.38%) when the number of mismatched base pairs allowed is 2. Although the sensitivity of Alientrimmer and Cutadapt is very high (100%), their extremely low specificity and accuracy indicate that they assume that all NGS reads have primers.

**Table 2:** Effects of mismatches on the performance of Alientrimmer, Cutadapt, cutPrimers and pTrimmer. with a simulation datasets (100×). Sensitivity (True Positive Rate, TPR) represent the proportion of reads whose primers were removed, in part or in full, by the programs in the reads that have primers introduced during simulation. Specificity (True Negative Rate, TNR) represent the proportion of reads for whom no primers were identified by the program in the reads that have no primers introduced during simulation. Accuracy (ACC) represent the proportion of reads whose primer sequences were precisely trimmed. Testing was performed with a simulation dataset of 100× depth with 1 ~ 5 mismatches allowed

Mismatch	Alientrimmer 1 threads, K = 9	Cutadapt 8 threads	cutPrimers 8 threads	pTrimer 2 threads, k = 9
	TPR (%)/TNR (%)/ACC (%)	TPR (%)/TNR (%)/ACC (%)	TPR (%)/TNR (%)/ACC (%)	TPR (%)/TNR (%)/ACC (%)
1	100.00/0.00/2.48	100.00/98.42/0.00	56.56/100.00/56.56	99.22/100.00/98.87
2	100.00/0.00/0.91	100.00/98.42/0.00	64.08/100.00/57.77	99.96/100.00/99.38
3	100.00/0.00/0.40	100.00/45.79/0.00	67.44/100.00/58.32	99.94/100.00/99.13
4	100.00/0.00/0.00	100.00/44.74/0.00	70.85/100.00/58.73	99.67/100.00/98.60
5	100.00/0.00/0.00	100.00/23.16/0.00	70.77/100.00/57.46	98.88/100.00/97.63

## Discussion

pTrimmer is a proper tool to trim off primer sequencing from MAS and target sequencing to ensure the reliable mutation landscape of download analysis. In addition, it also provided some useful function, such as parameter “--minqual” that could filter the reads by sequencing quality and “--seqtype” that enables to process both single-end and paired-end fastq file. A potential extension of pTrimmer is to remove adapter sequence by appropriately modifying the primer file. Except for the programs tested above, we also tested a bam-based primer trimming tools -- BAMClipper. Although the algorithm of BAMClipper is simple, we found its accuracy is comparable to pTrimmer. However, when the sequencing depth increases, its running time will increase rapidly (Fig. 3). In contrast, the running time of pTrimmer keeps almost unchanged even with 10x increases of NGS reads (Fig. 3).

## Conclusions

pTrimmer is a high performance and memory efficient multiplex amplicon sequencing primer trimmer. It is applicable to the study of ‘multiplex amplicon sequencing’ and ‘target sequencing’ that have multiple pairs of primers. The combinatorial algorithm of ‘k-mers model’ and ‘dynamic model’ ensures its high sensitivity and specificity. And the ‘normal condition’ and ‘read-through condition’ could make the pTrimmer have the ability to remove primers of both long and short DNA fragment. The ability to handle ‘read-through’ short reads makes pTrimmer be the ideal tool to process circulating tumor DNA (ctDNA)-based liquid biopsy data since ctDNA has the length of 132–145 bp that leads to read-through on regular Illumina platform [15].

### Availability and requirements

- Project name: pTrimmer
- Project home page: https://github.com/DMU-lilab/pTrimmer
- Operating system: Linux and Windows
- Programming language: C
- Other requirements: zlib-1.2.7 and gcc
- License: GNU General Public License v3.0
- Any restrictions to use by non-academics: GNU GPL License v3.0

## Additional files

Additional file 1: **Table S1.** The impact of primer regions on mutation calling. (DOCX 20 kb)

Additional file 2: **Figure S1.** Primer alignment algorithm, including both ‘k-mers model’ and ‘dynamic model’. The ‘k-mers model’ (line 8–17) will first try 12 times to find the target primer. If it fails, pTrimmer will perform a ‘dynamic model’ (line 20–35) to get the primer with the most k-mer hits. (PDF 13 kb)

## Abbreviations

cfDNA: Circulating cell-free DNA
ctDNA: Circulating tumor DNA.
MAS: Multiple amplicon-sequencing
NGS: Next generation sequencing
