# Supplementary material for: pTrimmer: An efficient tool to trim primers of multiplex deep sequencing data
Source: BMC Bioinformatics. 2019 May 10;20:236. doi: 10.1186/s12859-019-2854-x (PMC6511130; doi:10.1186/s12859-019-2854-x)
Supplement: Supplementary file 1 — Table S1. The impact of primer regions on mutation calling. (DOCX 20 kb) [file 12859_2019_2854_MOESM1_ESM.docx]

Additional file1

**Table S1. The impact of primer regions on mutation calling.** ‘Mutation1’ and ‘Mutation2’ represent the potential mutations called from raw reads and primer-trimed reads respectively. ‘Mutaion3’ represents the increased mutations caused by the inclusion of primers. And ‘Percentage’ is the increased mutation ratio due to the inclusion of primers. Raw reads were generated on Illumina X10 sequencing platform. Primers were trimmed by pTrimmer program. Potential mutations were called using Varscan by a single criteria of p value < 0.01. Stringent criterias were not adopted here in consideration that DNA synthesis errors are rare events. Raw reads denote the NGS read with primer

| Datasets | Mutation1 | Mutation2 | Mutation3 | Percentage (%) |
| --- | --- | --- | --- | --- |
| CfDNA1  CfDNA2  CfDNA3 | 8060  17378  54550 | 3892  9221  33760 | 4168  8157  20790 | 107.09  88.46  61.58 |

1. **Command Line for processing of CfDNA1-3**

**Datasets: CfDNAX indicate CfDNA1 CfDNA2 and CfDNA3**

**1.1 AlienTrimmer**

java -jar AlienTrimmer_0.4.0/src/AlienTrimmer.jar

-if Data/CfDNAX_R1.fq

-ir Data/CfDNAX_R2.fq

-c Alien_p/alien_CfDNA.fa # R1 5’-end primers and R2 5’-end primers

-of CfDNAX_trim_R1.fq

-or CfDNAX_trim_R2.fq

-os CfDNAX_single.fq

-k 9 -m 3

**1.2 Cutadapt**

cutadapt

-g file:cutadapt_p/5_primer.fa # R1 5’-end primers

-G file:cutadapt_p/5_primer.fa # R2 5’-end primers

-a file:cutadapt_p/3_primer.fa # reverse complementary of R2 5’-end primers

-A file:cutadapt_p/3_primer.fa # reverse complementary of R1 5’-end primers

-o CfDNAX_trim_R1.fq

-p CfDNAX_trim_R2.fq

Data/CfDNAX_R1.fq.gz"

Data/CfDNAX_R2.fq.gz"

-O 10 -e 0.1 -j 8 --pair-filter any

Note: python version ≥ 3.0

**1.3 cutPrimers**

python3 cutPrimer/cutPrimers-master/cutPrimers.py

--readsFile_r1 Data/CfDNAX_R1.fq.gz

--readsFile_r2 Data/CfDNAX_R2.fq.gz

--primersFileR1_5 cutPrimer_p/primer1_5.fa # R1 5’-end primers

--primersFileR1_3 cutPrimer_p/primer1_3.fa # reverse complementary of R2 5’-end primers

--primersFileR2_5 cutPrimer_p/primer2_5.fa # R2 5’-end primers

--primersFileR2_3 cutPrimer_p/primer2_3.fa # reverse complementary of R1 5’-end primers

--trimmedReadsR1 CfDNAX_trim_R1.fq

--trimmedReadsR2 CfDNAX_trim_R2.fq

--untrimmedReadsR1 CfDNAX_untrim_R1.fq

--untrimmedReadsR2 CfDNAX_untrim_R2.fq

--threads 8 --error-number 3

Note: python version ≥ 3.0

**1.4 pTrimers**

pTrimer-1.3.0

-s pair

-a pTrimer_p/CfDNA_primer.txt

-f Data/CfDNAX_R1.fq.gz

-r Data/CfDNAX_R2.fq.gz

-o output/

-k 9 -m 3 -l

**CfDNA_primer.txt format:**

ForwardPrimer ReversePrimer InsertLength AuxInfo (optional)

**Description:**

ForwardPrimer: R1 5’-end primers [R2 5’-end primers]

ReversePrimer: R2 5’-end primers [R1 5’-end primers]

InsertLength: the length of amplicon without primer sequence

AuxInfo: contain auxiliary information, eg. “KRAS chr12:25378499-25378527”
