# Supplementary material for: pTrimmer: An efficient tool to trim primers of multiplex deep sequencing data
Source: BMC Bioinformatics. 2019 May 10;20:236. doi: 10.1186/s12859-019-2854-x (PMC6511130; doi:10.1186/s12859-019-2854-x)
Supplement: Supplementary file 2 — Figure S1. Primer alignment algorithm, including both ‘k-mers model’ and ‘dynamic model’. The ‘k-mers model’ (line 8–17) will first try 12 times to find the target primer. If it fails, pTrimmer will perform a ‘dynamic model’ (line 20–35) to get the primer with the most k-mer hits. (PDF 13 kb) [file 12859_2019_2854_MOESM2_ESM.pdf]

```
kmer_length = 8
max_mismatch = 3     // maximum allowed mismatched base-pair
N = max_primer_length + 6
S = read_seq[0:N]
Hash = {all kmers}     // A hash table contains all kmer sequence of primers

/* start to perform 'k-mers model' */
i = 0
while i < 12 do
    key = S[i: i+kmer_length]
    primer = Hash[key]
    mis_num = Mismatch_Check(primer, S)
    if mis_num < max_mismatch then
        return primer
    end if
    i = i + 1
end while

/* if 'k-mers model' fails, start to perfrom 'dynamic model' */
K_Num = N - kmer_length      // k-mer number of sequence S
K = {k_j; j = 1..K_Num}           // all potential kmer sequence of S
Hit = {NULL}                        // record hit number for each canditate primer

j = 0
while j < K_Num do
    primer = Hash[K_j]
    Hit[primer] = Hit[primer] +1
    j = j + 1
end while

target_primer = Max_Hit_Primer(Hit)   // get primer that has highest kmer hit
mis_num = Dynamic_Check(target_primer, S) // Needleman-Wunsch local alignment
if mis_num < max_mismatch then
    return target_primer
end if
```
